# Somatosensory amplification and psychological distress in cancer survivors: the mediating role of fear of cancer recurrence

**DOI:** 10.1080/21642850.2025.2525184

**Published:** 2025-07-01

**Authors:** Shahaf Bitan, Shimrit Daches, Ilanit Hasson-Ohayon

**Affiliations:** Department of Psychology, Bar-Ilan University, Ramat-Gan, Israel

**Keywords:** Anxiety, cancer survivors, depression, fear of cancer recurrence, somatosensory amplification

## Abstract

**Introduction:**

Somatosensory amplification (SSA) refers to the tendency to experience body sensations as intense, noxious, and disturbing, and is associated with higher levels of anxiety and depression among cancer survivors. Body sensations among survivors are highly attributed to the possibility of cancer recurrence, thus triggering fear of cancer recurrence (FCR). Given that FCR itself is correlated with higher psychological distress, in this study we examined the mediating role of FCR in the relationship between SSA and symptoms of anxiety and depression among cancer survivors.

**Materials and Methods:**

One hundred and seventeen cancer survivors up to 10 years post-diagnosis took part in this cross-sectional study. Measures consisted of a demographic and medical questionnaire; the anxiety and depression domains of the Patient Reported Outcomes Measurement Information System (PROMIS); the Somatosensory Amplification Scale; and the Fear of Cancer Recurrence Inventory-Short Form. Mediation analyses were conducted to determine whether FCR mediated the relationships between SSA and both anxiety and depression.

**Results:**

SSA was found to be positively correlated with anxiety, depression, and FCR, and FCR was found to be positively correlated with anxiety and depression. FCR was found to fully mediate the relationships between SSA and both anxiety and depression.

**Conclusion:**

The findings suggest that FCR may serve as a mechanism linking SSA to psychological distress, highlighting the need to consider targeting FCR in interventions for cancer survivors.

## 1. Introduction

Advances in early detection, treatment, and demographic shifts have led to a growing number of cancer survivors (Miller et al., 2016). In the U.S., the survival rate for patients diagnosed between 2009 and 2015 reached 67% (Howlader et al., 2019), highlighting the need for research on post-cancer quality of life. Recovery extends beyond physical health, as survivors often struggle with returning to work and family life, and experience long-lasting emotional challenges (Laidsaar-Powell et al., 2019). A meta-analysis of 43 studies found that 17.9% of long-term survivors report heightened anxiety and 11.6% experience depression (Mitchell et al., 2013), with emotional distress lasting for extended periods (Lu et al., 2016; Zhao et al., 2014), disrupting daily life and reducing well-being (Wu & Harden, 2015). Consequently, exploring factors contributing to anxiety and depression in this population has become a major area of interest (Niedzwiedz et al., 2019). One such factor is somatosensory amplification (SSA) – a heightened sensitivity to bodily sensations (Carpenter et al., 2014; Igega & Carpenter, 2014).

Somatosensory amplification refers to the tendency to experience a somatic sensation as intense, noxious, and disturbing, and includes an individual's disposition to focus on unpleasant physiological sensations and consider them pathological rather than normal (Barsky, 1992; Barsky et al., 1988). According to Duddu et al. (2006), SSA includes three components: (1) bodily hyper-vigilance that involves heightened self-scrutiny and increased attention to unpleasant bodily sensations; (2) the tendency to select and focus on certain relatively weak or infrequent sensations; and (3) the tendency to cognitively appraise visceral and somatic sensations as abnormal, pathological, and symptomatic of disease, rather than as normal. This cognitive appraisal leads to alarm and anxiety over bodily sensations (Duddu et al., 2006). In the context of cancer, SSA levels were found to be significantly higher in patients with breast cancer compared to healthy controls (Kang et al., 2017), suggesting that the cancer experience is associated with excessive bodily alertness.

Given these characteristics, it is unsurprising that SSA has been consistently linked to heightened psychological distress. Research has demonstrated positive associations between SSA and anxiety and depression in both healthy individuals (Nakao et al., 2005) and those with medical conditions such as migraines (Yavuz et al., 2013) and acne (Öncü et al., 2022). Among patients with cancer undergoing treatment, higher SSA levels have been found to predict greater emotional distress, including elevated anxiety and depression (Cobeanu, 2013).

In cancer survivors, persistent monitoring, (mis)interpretation, and worry about uncertain bodily symptoms have been associated with increased anxiety and depression (Carpenter et al., 2014; Heathcote et al., 2023; Igega & Carpenter, 2014) as well as poorer health-related quality of life (Cunningham et al., 2021). However, while SSA has been found to be associated with anxiety and depression, the mechanisms underlying this relationship remain unclear. The Cancer Threat Interpretation Model proposed by Heathcote and Eccleston (2017) suggests that survivors, when experiencing somatic sensations, are prone to interpret these sensations as potentially threatening, particularly in the context of their cancer history. The model emphasizes how heightened sensitivity to bodily sensations (e.g. SSA) can amplify the appraisal of pain or discomfort as a sign of cancer recurrence, triggering maladaptive coping behaviors such as hyper-vigilance, misinterpretation, and anxiety. Given the uncertainty surrounding the recurrence of cancer, SSA may contribute to the emotional distress often observed in cancer survivors.

Fear of cancer recurrence (FCR) – the fear that cancer will return, progress, or metastasize – is considered one of the most distressing consequences of cancer, persisting even 10 years after diagnosis (Luigjes-Huizer et al., 2022; Simard et al., 2010). Previous studies have shown that cancer survivors who monitor their bodies and tend to interpret bodily sensations as threatening experience greater FCR (Heathcote et al., 2023; Verhoeven et al., 2005). In different literature, greater FCR has been associated with heightened psychological distress, including anxiety and depression (Götze et al., 2019; Liu et al., 2018). These findings nicely support the Cancer Threat Interpretation Model mentioned above (Heathcote & Eccleston, 2017), suggesting a possible mediating role of FCR in the relationship between SSA and anxiety and depression. To provide further support to this proposed model, the primary aim of the current study was to explore the relationships between SSA, FCR, and symptoms of anxiety and depression in a sample of cancer survivors. Specifically, this study examined whether FCR mediates the associations between SSA and both anxiety and depression.

Based on the reviewed literature, we hypothesized that (a) SSA would be positively correlated with both anxiety and depression, (b) SSA would be positively correlated with FCR, (c) FCR would be positively correlated with both anxiety and depression, and (d) the associations between SSA and both anxiety and depression would be mediated by FCR. Results of the current study will expand our knowledge about the possible underlying role that FCR plays in the association between SSA and psychological distress among cancer survivors, and will inform clinicians on possible correlates of anxiety and depression among this population.

## 2. Materials and methods

### 2.1. Participants and procedure

Participants (*N* = 117; 74.4% women) were survivors of a variety of cancer types, recruited between March-August 2023, either from a rehabilitation-focused mental health clinic for cancer survivors at Sheba Medical Center (21.4%) or from online survivor groups on social media (78.6%) in Israel. Survivors were eligible to participate if they met the following criteria: diagnosed with any type of cancer within the past 10 years, over 18 years of age at diagnosis, had completed active cancer treatment with curative intent and were currently cancer-free, and were able to complete an online survey in Hebrew.

Survivors were invited to participate in a research study on the psychological aspects of recovering from cancer. Those recruited through the medical center were informed about the study by a staff member, whereas those recruited through social media were informed by an ad posted on cancer survivor groups. The researchers contacted interested respondents by phone to explain the study in detail and confirm eligibility. Written informed consent was obtained from all 124 potential participants, who were sent a link for the online data collection forms. The anonymous online questionnaire, which took approximately 30 min to complete, was filled out by 119 potential participants (a 96% response rate for completing some or all of the questionnaire), after which they were thanked and paid for their participation (70 NIS, equivalent to 18.8 US dollars). Two potential participants' data were excluded because these individuals did not complete all the forms and did not meet the time-since-diagnosis eligibility criterion (according to the exact date of diagnosis reported in the questionnaire), resulting in a final sample of 117 participants.

An a priori power analysis was conducted using G*Power version 3.1.9.6 to determine the minimum sample size required to test the hypothesized mediation models (Faul et al., 2009). Results indicated that the required sample size needed to achieve 80% power for detecting an expected small to medium effect size, at a significance criterion of *α* = .05, was *N* = 113. Thus, the obtained sample size of *N* = 117 was adequate for testing the study hypotheses.

Ethics approval was obtained from both the Sheba Medical Center Ethics Committee (Approval number: SMC-9838-22) and the Bar-Ilan University Ethics Committee (Approval number: 2023/48).

### 2.2. Instruments

#### 2.2.1. Demographic and medical data questionnaire

Participants completed a questionnaire assessing personal characteristics (age, gender, marital status, years of education, and religiosity) and cancer-related medical information, including time since diagnosis, time since last treatment, cancer type, cancer stage, types of treatment received, and history of recurrence.

#### 2.2.2. Physical symptoms

Participants completed a set of items designed to assess the extent to which they experienced seven physical symptoms over the past week: pain, weakness, nausea, vomiting, constipation, diarrhea, and fatigue. These symptoms were selected based on previous research indicating their persistence during survivorship (Shi et al., 2011). Responses were recorded on a 4-point Likert-type scale, ranging from 1 (*not at all*) to 4 (*very much*), reflecting the extent to which participants had experienced each symptom. The items were summed to produce a total score ranging from 7 to 28, with higher scores indicating a greater overall symptom burden.

#### 2.2.3. Anxiety and depression

The anxiety and depression domains of the Patient Reported Outcomes Measurement Information System (PROMIS; Pilkonis et al., 2011) were used. Participants completed the PROMIS Short Forms for anxiety and depression, each consisting of eight items that assessed their experiences with common symptoms of anxiety or depression over the past week. Ratings were made on a 5-item Likert-type response scale, ranging from 1 (*not at all*) to 5 (*very much*). Scores were calculated according to the official scoring procedure manual of the National Institute of Health (NIH), such that a higher score indicates a higher symptom severity of anxiety or depression. A previous study provided support for the reliability and construct validity of the PROMIS anxiety and depression measures in a cancer population (Quach et al., 2016). In the current study, we used a validated Hebrew version of the questionnaire (Mosheva et al., 2020). Cronbach's alpha coefficients indicated excellent internal consistency in the current study (0.91 for anxiety and 0.93 for depression).

#### 2.2.4. SSA

The Somatosensory Amplification Scale (SSAS; Barsky et al., 1990) was used. The SSAS is a 10-item self-report questionnaire assessing the tendency to experience ordinary bodily and visceral sensations as intense, noxious, and disturbing. It includes a 5-item Likert-type response scale for participants to indicate the degree to which they experience different somatic and visceral sensations, ranging from 1 (*not at all true*) to 5 (*extremely true*). A higher average score of all items suggests greater symptom amplification, with scores ranging between 1 and 5. The scale has been found to have adequate internal consistency and test-retest reliability (Barsky et al., 1990; Cobeanu, 2013). The SSAS has been utilized in studies involving cancer survivors, though its specific validation within this population is limited (Barrineau et al., 2014; Carpenter et al., 2014). In the current study, the SSAS was translated into Hebrew using the standard translation and back-translation technique (Ohrbach et al., 2013). Cronbach's alpha coefficient indicated acceptable internal consistency in the current study (0.74).

#### 2.2.5. FCR

The Fear of Cancer Recurrence Inventory-Short Form (FCRI-SF; Simard & Savard, 2009, 2015) was used. The FCRI-SF is a 9-item self-report questionnaire assessing fear of recurrence. It includes a 5-item Likert-type response scale for participants to indicate the degree to which each item describes them, ranging from 0 (*not at all*) to 4 (*a great deal*). The nine items are summed up into a total score ranging between 0 and 36, so that a higher score suggests a greater FCR. The FCRI-SF, which is the severity subscale of the FCRI, is highly correlated with the FCRI total score (*r* = 0.84), has good internal consistency (*r* = 0.89) and test-retest reliability over one month (*r* = 0.80), and demonstrates convergent validity with other FCR measures (Simard & Savard, 2009). In the current study, we used a validated Hebrew version of the FCRI-SF (Bentley et al., 2023). Cronbach's alpha coefficient of internal consistency in the current study was good (0.85).

### 2.3. Statistical analysis

Data collected from the sample of 117 participants were analyzed using jamovi version 2.3 software (2023). Descriptive statistics were computed for demographic and medical characteristics and for key variables of interest. Pearson correlation coefficients were calculated to examine bivariate associations among study variables. Linear regression and hierarchical regression analyses were used to evaluate the total (c path), direct (c′ path), and indirect effects. Assumptions of normality and homoscedasticity were checked for all regression analyses, and no violations were found. Mediation analyses were conducted using the jAMM module (jamovi advanced mediation models module; Gallucci, 2020), which implements bootstrapped regression procedures (5000 samples) to estimate indirect effects of SSA on psychological distress (anxiety, depression) through FCR. Given that age and time since diagnosis were the only sociodemographic and clinical variables significantly associated with key study variables, we explored their potential influence by including them as covariates in supplementary mediation models.

## 3. Results

### 3.1. Demographic and medical characteristics

Table 1 presents participants' sociodemographic and medical characteristics. Of the 117 cancer survivors, 87 were women (74.4%). Participants' average age was 47 years (*SD* = 13.4, range 20–72), and most were married and secular. The average time since diagnosis was 37.1 months (*SD* = 26.2, range 4–116), and most had had breast cancer (43.6%), lymphoma (20.5%), or gastrointestinal cancer (18.8%). Moreover, most of them had had Stage II or III cancer, and had undergone chemotherapy, surgery, and/or radiation therapy. The average physical symptoms level reported by the participants was 13.9 (*SD* = 4.44, range 7–27, median = 13). In addition, 10 participants (8.5%) had a history of cancer recurrence (most of whom were men; *n* = 6).

**Table 1.** Demographic and medical characteristics of participants (*N* = 117).

Variable	Mean	*SD*	Range
Age	47	13.4	20–72
Years of education	15.2	3.06	9–26
Time since diagnosis (months)	37.1	26.2	4–116
Time since last treatment (months)	26.1	23.9	1–96
Physical symptoms scale score	13.9	4.44	7–27
Variable	Category	*N*	%
Gender	Female	87	74.4
	Male	30	25.6
Marital status	Married	67	57.3
	Divorced	24	20.5
	Single	24	20.5
	Widowed	2	1.7
Religiosity	Secular	66	56.4
	Traditional	29	24.8
	Religious	22	18.8
Cancer type	Breast	51	43.6
	Lymphoma	24	20.5
	Gastrointestinal	22	18.8
	Testicular	5	4.3
	Leukemia	4	3.4
	Lung	4	3.4
	Gynecological	3	2.6
	Others	4	3.4
Cancer stage	0	3	2.6
	I	12	10.3
	II	44	37.6
	III	35	29.9
	IV	23	19.7
Cancer treatment	Chemotherapy	96	82.1
	Surgery	79	67.5
	Radiation therapy	72	61.5
	Biological therapy	34	29.1
	Hormone therapy	16	13.7
Recurrence	No	107	91.5
	Yes	10	8.5

### 3.2. Correlations between anxiety, depression, SSA, and FCR

Correlations between anxiety, depression, SSA, and FCR are presented in Table 2. In line with our first hypothesis, SSA was significantly and positively correlated with both anxiety (95% CI [.079, .419], *p* = .005) and depression (95% CI [.038, .385], *p* = .018). In line with our second hypothesis, SSA was significantly and positively correlated with FCR (95% CI [.112, .446], *p* = .002). We also calculated the partial correlation between SSA and FCR, controlling for the physical symptoms scale score, which revealed a significant positive correlation (*r* = .233, 95% CI [.053, .415], *p* = .012). Moreover, in line with our third hypothesis, FCR was significantly and positively correlated with both anxiety (95% CI [.281, .576], *p* < .001) and depression (95% CI [.1, .436], *p* = .003). Out of the sociodemographic and clinical variables examined, only age was significantly correlated with depression, SSA, and FCR, while time since diagnosis was significantly correlated with FCR (see Table 2). Other variables – including gender, recurrence history, cancer type, and cancer stage – were not significantly associated with the key study variables (all *p* > .05; assessed via *t*-tests, ANOVAs, and Spearman correlations as appropriate).

**Table 2.** Descriptive statistics and bivariate correlations between the study variables (*N* = 117).

	Mean	SD	Range	Anxiety	Depression	SSA	FCR	Age
Anxiety	62.1	6.69	43.2–80	–				
Depression	56.2	8.5	37.1–81.1	0.691***	–			
SSA	2.8	0.69	1.4–4.7	0.257**	0.218*	–		
FCR	19.7	6.89	4–36	0.44***	0.277**	0.287**	–	
Age	47	13.4	20–72	−0.143	−0.211*	−0.255**	−0.24**	–
Time since diagnosis (months)	37.1	26.2	4–116	−0.091	−0.062	−0.01	−0.245**	0.331***

Abbreviations: FCR, fear of cancer recurrence; SSA, somatosensory amplification.

* *p* < .05, ** *p* < .01, *** *p* < .001

### 3.3. Mediation models

In order to test our fourth hypothesis – namely, that the relationships between SSA and anxiety and depression would be mediated by FCR – we explored two different mediation models: the first one with SSA as the independent variable, FCR as the mediator, and anxiety as the dependent variable, and the second one with SSA as the independent variable, FCR as the mediator, and depression as the dependent variable. Subsequently, these mediation models were re-run controlling for potential covariates – age and time since diagnosis – which were the only sociodemographic and clinical variables significantly correlated with key study variables.

Within the full regression of the first model, in which anxiety was the dependent variable, 21.2% of the variance ratings of anxiety were explained by the combination of SSA and FCR, *R*^2^ = .21. Adj. *R*^2^ = .20. This finding represents a significant amount of the explained variance, *F*(2, 114) = 15.37, *p* < .001.

**Figure 1.**
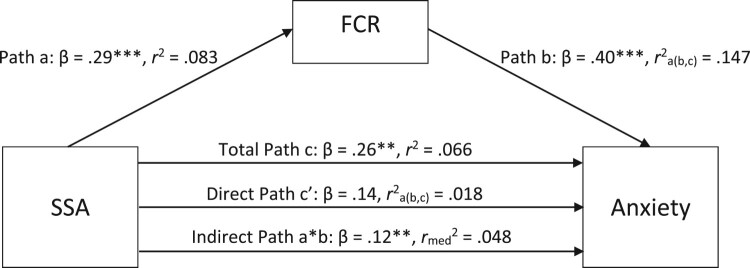
Standardized regression coefficients and squared correlation effect sizes for the relationship between somatosensory amplification (SSA) and anxiety, as mediated by fear of cancer recurrence (FCR). * *p* < .05, ** *p* < .01, *** *p* < .001.

Using 5000 bootstrapped samples, the indirect effect was significant, providing statistical support for the argument that mediation was present, *B* = 1.11, 95% BS*p* CI [.39, 2.03], *β* = .12. The direct effect of SSA on anxiety was not significant, *B* = 1.37, 95% BS*p* CI [−.03, 2.82], *β* = .14, indicating that the mediation effect was full. Figure 1 below provides a visual representation of the various pathways of the mediation model when the dependent variable was anxiety.

Within the full regression of the second model, in which depression was the dependent variable, 9.8% of the variance ratings of depression were explained by the combination of SSA and FCR, *R*^2^ = .10. Adj. *R*^2^ = .08. This finding represents a significant amount of the explained variance, *F*(2, 114) = 6.16, *p* < .01.

**Figure 2.**
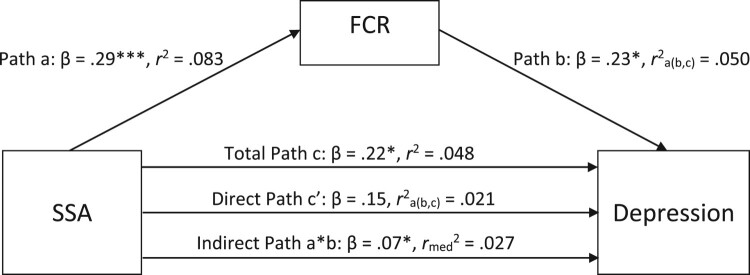
Standardized regression coefficients and squared correlation effect sizes for the relationship between somatosensory amplification (SSA) and depression, as mediated by fear of cancer recurrence (FCR). * *p* < .05, ** *p* < .01, *** *p* < .001.

Using 5000 bootstrapped samples, the indirect effect was significant, providing statistical support for the argument that mediation was present, *B* = .82, 95% BS*p* CI [.14, 1.71], *β* = .07. The direct effect of SSA on depression was not significant, *B* = 1.86, 95% BS*p* CI [−.63, 4.41], *β* = .15, indicating that the mediation effect was full. Figure 2 below provides a visual representation of the various pathways of the mediation model when the dependent variable was depression.

Notably, when including age and time since diagnosis as covariates in supplementary analyses, results remained largely unchanged. However, in the model predicting depression, the indirect effect of SSA on depression through FCR was no longer statistically significant, *B* = .69, 95% BS*p* CI [0.09, 1.49], *β* = .06, *p* = .07.

## 4. Discussion

In the current study, we examined the role of FCR as a possible mediator in the relationship between SSA and symptoms of anxiety and depression among cancer survivors. In line with our first hypothesis, SSA was found to be positively correlated with both anxiety and depression, suggesting that survivors who are more vigilant for bodily symptoms, and who are more likely to interpret them as pathological, tend to experience higher levels of psychological distress. This finding is consistent with previous studies on cancer survivors, showing that monitoring, (mis)interpreting, and worrying about uncertain bodily symptoms have been linked to heightened anxiety and depression (Carpenter et al., 2014; Heathcote et al., 2023; Igega & Carpenter, 2014). There is an association between the threatening and exhausting physical experience that patients in the acute phase of cancer undergo and excessive alertness to their bodily symptoms (Kang et al., 2017), and this kind of hyper-vigilance continues to be prominent in cancer survivors' day-to-day experience as well (Mutsaers et al., 2016). Our study provides further evidence that this excessive alertness to the body in cancer-free survivors following treatment is associated with higher levels of psychological distress.

In line with our second hypothesis, SSA was found to be positively correlated with FCR, suggesting that survivors who are more vigilant for bodily symptoms and who are more likely to interpret them as pathological tend to experience higher levels of FCR. Notably, this association remained significant even after controlling for current physical symptoms, underscoring the independent effect of SSA on FCR. This finding aligns with previous research showing that survivors who closely monitor their bodies and perceive bodily sensations as threatening are more likely to experience heightened FCR (Heathcote et al., 2023; Verhoeven et al., 2005). This might be because amplified bodily sensations are interpreted as threatening signs of cancer recurrence (Tuman et al., 2021). Furthermore, in line with our third hypothesis, FCR was found to be positively correlated with both anxiety and depression, strengthening the previously well-established evidence that survivors who experience greater FCR experience higher levels of anxiety and depression as well (Götze et al., 2019; Liu et al., 2018).

Beyond these correlative findings, the current study expands the existing literature by showing that in line with our fourth hypothesis, the associations between SSA and both anxiety and depression were found to be fully mediated by FCR. The mediation models suggest that survivors who tend to focus on unpleasant somatic sensations and to experience them as intense, noxious, and disturbing experience greater FCR, which in turn is associated with higher levels of anxiety and depression. That is, excessive and fearful engagement with the possibility that cancer will return may explain the association between excessive engagement with the body and psychological distress among cancer survivors.

As suggested by the Cancer Threat Interpretation Model (Heathcote & Eccleston, 2017), the current study supports the idea that somatic sensations, particularly those that are amplified, may be perceived as threatening due to the uncertainty and fear of disease recurrence. The mediating role of FCR further associates heightened attention to bodily sensations with increased anxiety and depression. Specifically, survivors with elevated SSA may interpret their bodily symptoms as indicators of potential cancer recurrence, which is associated with greater FCR that, in turn, may contribute to higher levels of psychological distress. These findings, together with the Cancer Threat Interpretation Model, suggest that the emotional well-being of survivors is associated with how they attribute amplified bodily symptoms – whether to cancer threat or other non-threatening causes.

Future research should further explore the complex relationships between SSA, FCR, and distress. Longitudinal studies could shed light on the temporal dynamics between these variables, and qualitative investigations could offer a deeper understanding of the mediation models proposed in this study. Additionally, examining SSA, FCR, and psychological distress in more homogeneous samples based on time since diagnosis and cancer type – key variables for FCR (Götze et al., 2019; Simard et al., 2010) – would provide valuable insights. In the current study, the indirect effect of SSA on depression through FCR was no longer statistically significant when age and time since diagnosis were controlled for, suggesting that these variables may moderate this pathway. This may reflect the broader influence of age and time since diagnosis on how survivors perceive bodily sensations and cope with the ongoing threat of cancer, including possible shifts in health vigilance, emotional reactivity, and psychological adjustment over time. Future research should investigate how these demographic and illness-related factors influence the strength and stability of cognitive–affective mechanisms such as the SSA-FCR-distress pathway. It would also be worthwhile to assess the potential vicious cycle between SSA and FCR, which may amplify somatic symptoms on one hand and exacerbate anxiety and depression symptoms on the other (Heathcote & Eccleston, 2017).

### 4.1. Study limitations

Our findings should be considered in light of certain limitations. First, due to the cross-sectional nature of the study, causality cannot be determined, and changes in FCR, anxiety, or depression over time could not be detected. Second, the sample in the current study was highly heterogeneous with regard to time since diagnosis and types of cancer. Third, due to the higher responsiveness of women than men to the invitation to participate in the study, the sample was not optimally balanced in terms of gender representation. In this regard, we decided to include participants who had a history of cancer recurrence despite their small number, given that most of them were men, and thus male representation was improved in the sample. While all participants were cancer-free at the time of the study, a history of recurrence may be associated with heightened symptom monitoring, which could influence SSA scores. Although our analyses revealed no significant associations between a history of recurrence and the key study variables, the small size of this subgroup limits the ability to detect such effects. Fourth, while SSAS was validated in other populations, further research is necessary to confirm its validity in cancer survivor populations. This limitation raises the possibility that the measure may not fully capture the construct of SSA as experienced in this specific group, potentially affecting the precision and generalizability of the findings. Finally, although supplementary analyses controlling for age and time since diagnosis had minimal impact overall, they did attenuate the indirect effect of SSA on depression. This highlights the need to consider these variables when interpreting mediation effects.

### 4.2. Clinical implications

The current findings have clinical implications for therapeutic interventions aimed at improving coping with anxiety and depression among cancer survivors. Our results suggest that survivors who focus excessively on bodily symptoms may be at heightened risk for emotional distress. Cognitive behavioral therapy, which has demonstrated effectiveness in addressing body hyper-vigilance and somatic amplification (Barsky & Silbersweig, 2023), may thus be a valuable treatment option for survivors with high SSA. However, the mediation model proposed in this study highlights the need to consider FCR as a key factor in managing distress in this population. Instead of discouraging symptom monitoring – since this could potentially lead to missing early signs of recurrence – interventions could benefit from helping survivors expand their interpretations of bodily sensations, moving beyond the catastrophic interpretation of recurrence. Consistent with this approach, Cillessen et al. (2018) demonstrated that reductions in FCR mediated improvements in anxiety and depression following mindfulness-based cognitive therapy. A systematic review and meta-analysis by Lyu et al. (2022) provides further guidance for clinicians, indicating that face-to-face, short-term, mindfulness and acceptance-based interventions are effective in reducing FCR.

## 5. Conclusions

The current cross-sectional study expands the existing literature by showing that FCR may explain the associations between SSA and both anxiety and depression among cancer survivors. The results suggest that cancer survivors who experience amplified somatic sensations may attribute them to a potential cancer recurrence, which is in turn associated with higher psychological distress. Therefore, our results highlight the need to target FCR in interventions for survivors with high SSA.

## Data Availability

The data that support the findings of this study are available from the corresponding author upon reasonable request.
